# Reprofiling using a zebrafish melanoma model reveals drugs cooperating with targeted therapeutics

**DOI:** 10.18632/oncotarget.9613

**Published:** 2016-05-26

**Authors:** Laura Fernandez del Ama, Mary Jones, Paul Walker, Anna Chapman, Julia A. Braun, Jasmine Mohr, Adam F. L. Hurlstone

**Affiliations:** ^1^ Faculty of Life Sciences, The University of Manchester, Manchester, UK

**Keywords:** melanoma, zebrafish, MEK, PI3K, mTOR

## Abstract

Phenotype-guided re-profiling of approved drug molecules presents an accelerated route to developing anticancer therapeutics by bypassing the target-identification bottleneck of target-based approaches and by sampling drugs already in the clinic. Further, combinations incorporating targeted therapies can be screened for both efficacy and toxicity. Previously we have developed an oncogenic-RAS-driven zebrafish melanoma model that we now describe display melanocyte hyperplasia while still embryos. Having devised a rapid method for quantifying melanocyte burden, we show that this phenotype can be chemically suppressed by incubating V12RAS transgenic embryos with potent and selective small molecule inhibitors of either MEK or PI3K/mTOR. Moreover, we demonstrate that combining MEK inhibitors (MEKi) with dual PI3K/mTOR inhibitors (PI3K/mTORi) resulted in a super-additive suppression of melanocyte hyperplasia. The robustness and simplicity of our novel screening assay inspired us to perform a modest screen of FDA approved compounds for their ability to potentiate MEKi PD184352 or PI3K/mTORi NVPBEZ235 suppression of V12RAS-driven melanocyte hyperplasia. Through this route, we confirmed Rapamycin as a compound that could synergize with MEKi and even more so with PI3K/mTORi to suppress melanoma development, including suppressing the growth of cultured human melanoma cells. Further, we discovered two additional compounds—Disulfiram and Tanshinone—that also co-operate with MEKi to suppress the growth of transformed zebrafish melanocytes and showed activity toward cultured human melanoma cells. In conclusion, we provide proof-of-concept that our phenotype-guided screen could be used to identify compounds that affect melanoma development and prompt further evaluation of Disulfiram and Tanshinone as possible partners for combination therapy.

## INTRODUCTION

Cutaneous melanoma is the deadliest form of skin cancer, accounting for 80% of skin-cancer related deaths. It arises from melanocytes, pigmented epidermal cells. Worldwide, melanoma incidence keeps increasing. At early stages of melanoma, the prognosis is good as treatment, involving surgical excision, is usually highly effective. However, in the vertical growth phase (VGP), when melanoma invades subcutaneous tissue, and in the metastatic stages, it is largely incurable. Initiation of melanocyte neoplasia requires activation of ERK MAPK signalling, typically achieved by mutational activation of BRAF or NRAS or through mutational inactivation of NF1 [1]. Simultaneous activation of the ERK MAPK and PI3K-AKT-mTOR pathways is required for progression to malignancy [2, 3].

Monotherapies typically have low efficacy for treating disseminated cancer. To increase the efficacy of targeted therapy and obviate resistance, multiple signalling pathways need to be targeted [3–5]. Drug synergy also permits reducing the dosage of each agent, and thereby the toxicity from on- and off-target effects [6]. Multiple strategies exist for identifying effective combinations of drugs, with the most widely applied involving in vitro high-throughput screening (HTS) where large collections of molecules are tested using biochemical or cell-based assays. Other projects select fewer agents based on their known targets and use in vivo assays to guide selection. In vivo HTS are rarely done because of the cost and time entailed screening relevant animal, for example mammalian, models. On the other hand, given the characteristics of zebrafish, and the high genetic and physiological similarities between them and humans, this increasingly popular model can be used as a useful and cost-effective vehicle for HTS [7].

We have previously reported on a transgenic zebrafish line—Tg(mitfa:V12HRAS; mitfa:GFP)— expressing oncogenic HRAS^G12V^ in their melanocytes (herein named V12RAS fish) that present with radial growth phase (RGP) melanoma and occasionally develop raised tumour nodules, accompanied by more invasive growth, which we equate to vertical growth phase (VGP) melanoma [2]. This zebrafish melanoma model also displays activation of both ERK MAPK and PI3K-AKT signalling pathways [2]. In the present work, we demonstrate that even as embryos these transgenic zebrafish present with increased melanocytes and consequently more melanin which we exploit to assay small molecules for their ability to modulate the development of melanoma. We not only demonstrate that MEKi and dual PI3K/mTORi can supress V12RAS-driven melanocytic hyperplasia, but we also confirm synergism when inhibitors were combined. Based on this, we scaled up the procedure in a HTS-like setting in order to screen a library of 640 FDA approved small molecules in combination with either PD184352 (a MEKi) or NVPBEZ235 (a dual PI3K/MTORi). The screen recovered a number of hits among which Rapamycin, an mTOR inhibitor with proven antineoplastic activity when in combination, showed potent synergy with NVPBEZ235, providing a proof-of-principle that the screen could find effective combinations of antineoplastic agents. Disulfiram and Tanshinone were also revealed to possess anti-melanoma activity.

## RESULTS

### A zebrafish larval model of melanoma onset

**Figure 1:**
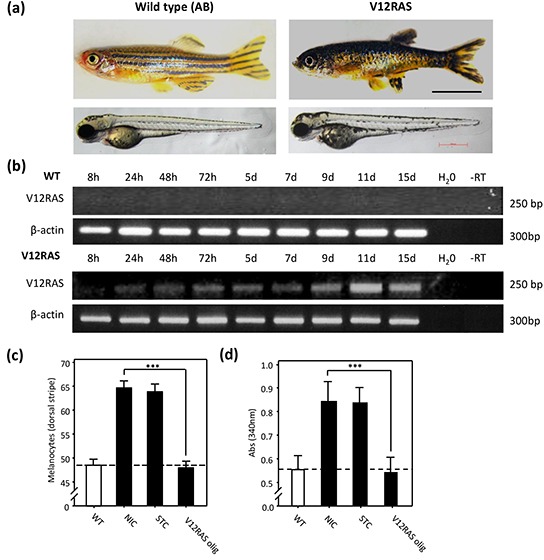
Melanocyte hyplerplasia in V12HRAS-expressing zebrafish embryos **a.** Upper pictures: WT (left) and V12RAS (right) transgenic adult fish. Lower pictures: 5dpf embryos. **b.** Expression of exogenous V12RAS detected by RT-PCR at different time points. β-catenin is a control for cDNA integrity. **c.** Quantification of the number of melanocytes in thirty wild-type (WT) or non-injected control (NIC), standard control oligonucleotide (STC)-injected or antisense oligonucleotide targeting exogenous V12RAS (V12RAS olig)-injected 5dpf V12RAS transgenic embryos. Shown are means ±SEM. **d.** Quantification of the melanin absorbance (λ= 340nm) for eight wild-type (WT) or NIC, STC-injected and V12RASolig-injected 5dpf V12RAS transgenic embryos. Shown are means ±SEM for six independent experiments. (c) and (d) For easier reference, the dashed line indicates the mean value for wild-type embryos. *** P<0.001 independent samples t-test.

When compared to wild-type subjects, adult V12RAS transgenic zebrafish display a distinctive phenotype, characterised by aberrant hyper-pigmentation (Figure 1a, upper pictures) (see also [2]). Moreover, the onset of this adult phenotype was already apparent in V12RAS larvae (Figure 1a, lower pictures) (see also [8]). We wanted to verify that transgenic larvae were in fact over-expressing HRAS^G12V^. Reverse Transcription coupled-Polymerase Chain Reaction (RT-PCR) revealed that HRAS^G12V^ is expressed in transgenic larvae from 24hpf (hours post-fertilization) onwards, but as expected no expression was detected in wild-type larvae (Figure 1b). Counting revealed a 30% increase in melanocyte numbers in transgenic larvae by 5 days post-fertilization (dpf) (Figure 1c) (see also [8]). Knockdown of HRAS^G12V^ using a morpholino-modified antisense oligonucleotide targeting the HA-tag translation start codon prevented excess melanocyte proliferation (Figure 1c), consistent with the observed hyperplasia stemming from the action of oncogenic RAS in melanocytes. We next developed a simple assay for automated quantitation of pigment as a corollary of hyperplasia in transgenic larvae (see also [8]). Melanin was extracted from embryos at 5dpf and the absorbance of lysates determined at 340 nm. Absorbance from V12RAS transgenic larvae was 1.43 fold greater than for wildtype larvae (Figure 1d) (see also [8]). Furthermore, using melanin content we were also able to confirm that knockdown of HRAS^G12V^ suppressed RAS-driven hyperplasia in transgenic larvae (Figure 1d).

### Suppression of V12RAS-driven melanocyte hyperplasia by MEKi and PI3K/mTORi

**Figure 2:**
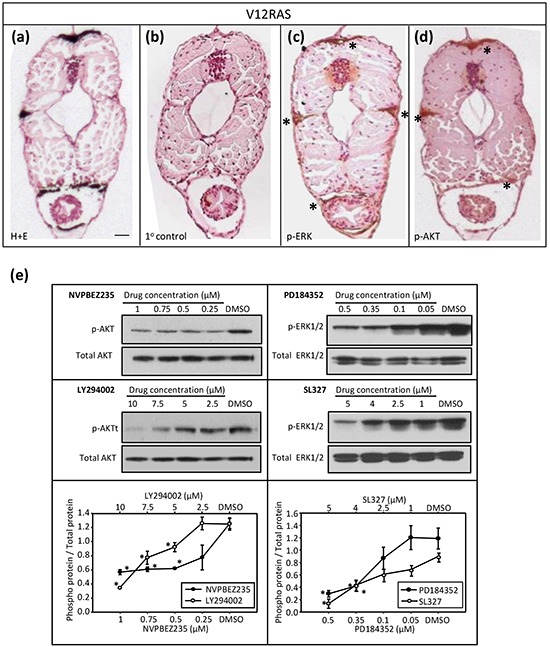
Molecular characterization of the V12RAS model **a–d.** Transverse sections of V12RAS larvae. (a) H&E staining. Scale bar = 0.2 mm. **(b–d)** IHC on bleached sections. (b) Non-specific primary control, (c) phospho-ERK and (d) phospho-Akt. Positive stain (brown) denoted by asterisks. **e.** Results of immunoblotting protein extract (30 μg) from 30 pooled 5-dpf embryos exposed for 4 h to the specified drug at the indicated concentrations. Representative immunoblots are depicted with densitometric quantification shown immediately below (mean p-protein/total protein ± SEM for three independent experiments). *P<0.05 compared to vehicle control; independent samples t-test.

**Figure 3:**
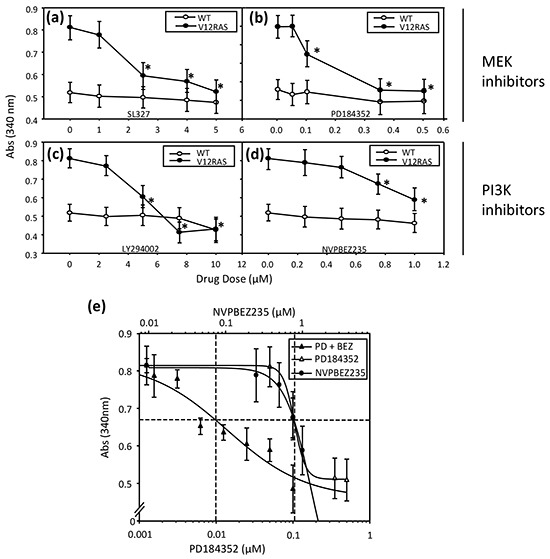
Dose dependent suppression of V12RAS-driven melanocyte hyplerplasia using selected MEKi and PI3K/mTORi 2-dpf V12RAS and WT embryos were exposed for 72h to the indicated concentrations of MEKi—**a.** SL327 and **b.** PD184352— and dual PI3K/mTORi—**c.** LY294002 and **d.** NVPBEZ235. Melanin content was then determined. Shown are means ± SEM (n = 6 wells). *P<0.05 compared to vehicle control; independent samples t-test. **e.** 2-dpf V12RAS were exposed for 72h to PD184352 or NVPBEZ235 alone or in combination followed by determination of melanin content.

Previously, we have used immunohistochemistry (IHC) to confirm activation of ERK MAPK and PI3K-AKT signalling in zebrafish melanoma resulting from expression of HRAS^G12V^ [2]. IHC on transverse sections of V12RAS larvae also revealed activated, phosphorylated (p)-ERK and p-AKT in the melanocytes (Figure 2a–2d), indicating that these signaling modules are hyper-activated at this early stage, as later in tumor nodules. Next we established that incubating 5dpf zebrafish larvae for 4h with highly potent and selective MEKi (SL327 and PD184352) or PI3K/mTORi (NVPBEZ235 and LY294002) resulted in a dose-dependent reduction in phosphorylated ERK and AKT (Figure 2e). Moreover, incubating V12RAS transgenic larvae with these inhibitors between 2 and 5 dpf resulted in a reduction of melanocyte hyperplasia as assessed by melanin content, but only at doses capable of suppressing ERK and AKT phosphorylation (Figure 3a–3d). Further, to evaluate a potential synergy from targeting simultaneously ERK MAPK and PI3K pathways, larvae were treated with a combination of NVPBEZ235 and PD184352. The drugs were combined in a ratio corresponding to the concentrations required for each separate drug to suppress excess melanin by 50%. Mixtures were then serially diluted to allow dose-dependency to be evaluated. The combination showed an enhanced ability to selectively suppress melanocyte hyperplasia in V12RAS-expressing larvae compared to the individual component drugs (Figure 3e). No effect on wild-type melanin levels was observed for this mixture over a range of efficacious concentrations (data not shown), demonstrating that the combination was well tolerated.

### HTS using an FDA library

**Figure 4:**
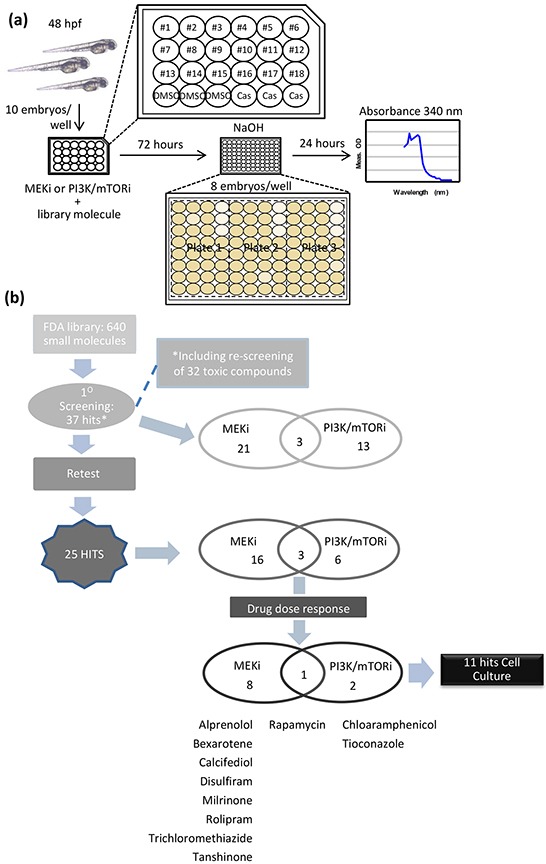
Screen design and Hit selection summary **a.** Schematic depicting the steps involved in screening the FDA-library. **b.** Schematic summarizing the hits identified after the screening procedure, retesting using 5 replicates, and drug dose response using the melanin assay. At the end of this process there were 11 hits to be further evaluated in cell culture.

Using the pilot data above, we were able to determine a Z-factor (Z') = 0.6 for the melanin absorption assay to detect the ability of small molecule inhibitors to suppress melanocyte hyperplasia in V12RAS transgenic embryos. Based on this value, which indicates a high degree of discrimination [9], we scaled up embryo production in order to screen 640 small molecules from a FDA approved library of biologically active compounds in combination with either sub-optimal doses of PD184352 (0.1 μM) or NVPBEZ235 (0.3 μM). Using the process outlined in Figure 4a, ten hatched 2dpf V12RAS embryos were incubated in a 24-well plate with the aforementioned combinations of drugs. A single well was used per compound. V12RAS transgenic animals treated with the vehicle control 0.1% dimethyl sulfoxide (DMSO) and *Casper* embryos were included on each plate for internal calibration and normalisation (see Figure 4a for layout of plate). 72 hours later, eight embryos were transferred to a 96-well plate where they were dissolved for absorbance reading at 340nm. At this stage, this process also allowed us to determine drug toxicity, since drugs would only be included in the analysis if 80% or more of embryos survived the treatment, and the embryos did not show any behavioural or morphological defects of developmental toxicity as previously described [10]. Toxic drugs were rescreened at half the starting concentration (an average of 1 μM). Absorbance for *Casper* embryos, which only have melanin in the pigmented retinal epithelium, was subtracted from the V12RAS embryo values to correct for this background signal. Subtracted values from each well were then normalized to values obtained from wells containing DMSO-treated V12RAS transgenic animals.

**Figure 5:**
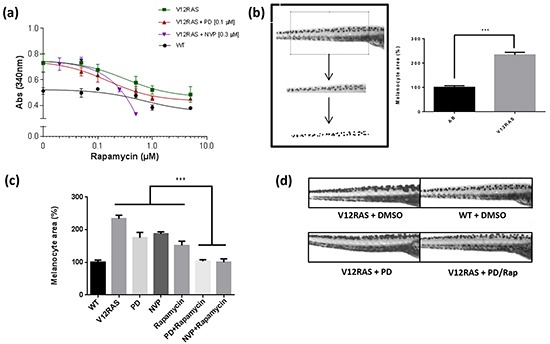

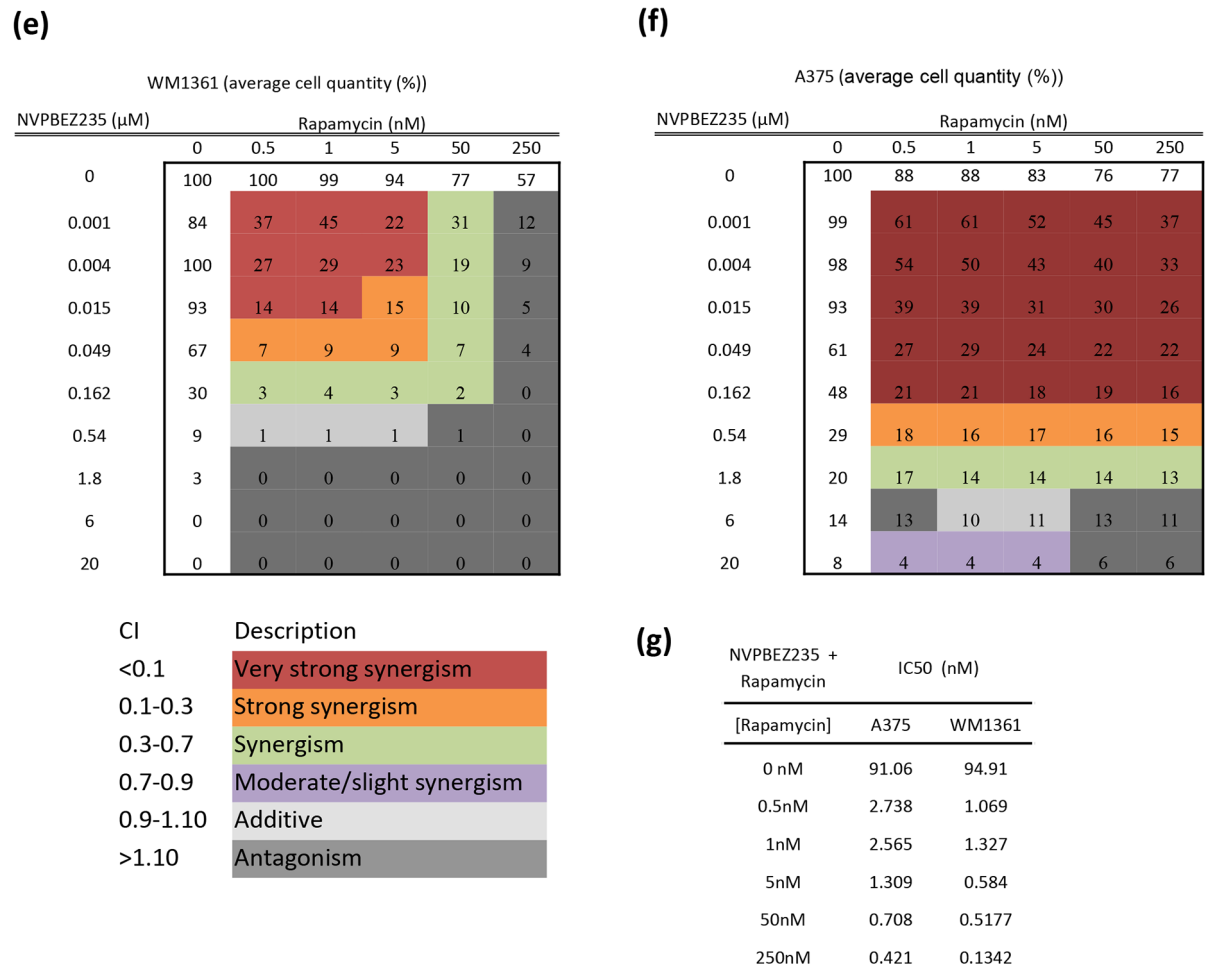
Synergistic effect of Rapamycin **a.** In-vivo drug dose curve for Rapamycin using the melanin assay. Rapamycin was tested at different concentrations in V12RAS and WT embryos, and in combination with MEK and mTOR/PI3K inhibitors in the transgenic embryos. Points depict mean ± SEM for three independent experiments. **b.** Imaging-based method for determining melanocyte burden. Values shown are mean pigmented areas ± SEM for thirty embryos *** P<0.001 independent samples t-test. **c.** Melanocyte area quantification in V12RAS embryos treated with PD184352 (PD) (0.1 μM), NVPBEZ235 (NVP) (0.3 μM), Rapamycin (1 μM), Rapamycin (1 μM) in combination with PD (0.1 μM), and Rapamycin (0.25 μM) in combination with NVP (0.3 μM). *** P<0.001 One-way ANOVA. **d.** Lateral views of 5dpf zebrafish embryos from the yolk sac to nearly the end of the tail treated as indicated. Average cell number (%) measured in **e.** WM1361 and **f.** A375 cell lines when treated with different combinations of Rapamycin and NVPBEZ235 concentrations for 72h. The colour code corresponds to the Chou-Talalay combination index (CI) used to quantify the level of synergism. **g.** The corresponding IC_50_ values for the above cell line treatments.

Forty-eight compounds in combination with either PD184352 or NVPBEZ235 were screened per week by a single investigator. Having screened the library of 640 molecules, the median normalized melanin absorbance was calculated. Hits were then selected following the median and median absolute deviation (MAD) method [11, 12] with a cut-off established at −2.5MAD (Supplementary Figure S1), giving as a result 37 primary hits. Following repetition of the screening assay but now on 5 wells, this number was reduced to 25 compounds capable of suppressing the V12RAS phenotype (Supplementary Figure S2a and Figure 4b). To qualify for further evaluation, hits were examined for dose-dependency and their influence on pigmentation of wild-type animals. Based on clear dose-dependency, co-operation with either PD184352 or NVPBEZ235 (that is a stronger effect of the combination than library compound alone), and negligible effect on pigmentation of wild-type animals, only 11 hits were selected for further assessment (listed in Figure 4b). Dose-response curves are shown for Rapamycin (Figure 5a) as well as the other 10 shortlisted hits (Supplementary Figure S3). L-Thyroxine, which emerged as the most potent hit from the primary screen (Supplementary Figure S1 and S2a), didn't exhibit co-operation at any dose and also completely suppressed pigmentation in wild-type zebrafish (Supplementary Figure S3) and was excluded from subsequent evaluation for this reason. Other compounds (for example Fluvastatin) possessed significant single-agent activity which was selective for V12RAS transgenic larvae but showed only a subtle co-operative effect, so were also excluded (data not shown).

### Rapamycin: an example of a hit demonstrating clear synergism

Among the hits we identified, the mTOR inhibitor Rapamycin showed a strong effect in combination with PD184352 and the strongest effect of all compounds tested (excluding L Thyroxine) with the dual PI3K/MTOR inhibitor NVPBEZ235 (Figure 5a). In order to determine whether this result was really due to a suppression of V12RAS-driven melanocyte hyperplasia and not just an effect on melanin production, we devised another means of quantifying the melanocyte burden of larvae. Images of the dorsal stripe were acquired at 5dpf, a region of interest fitted to the portion of stripe overlying the yolk sac extension, and a binary mask applied using ImageJ to nullify potential differences in melanin levels per melanocyte. The area of pigmentation, proportional to the number of melanocytes, was then quantified using ImageJ. This analysis verified a consistent and significant increase of melanocyte burden in V12RAS embryos compared to WT embryos (Figure 5b). Moreover, while treatment of V12RAS with low dose PD184352, NVPBEZ235 or Rapamycin did not affect significantly melanocyte burden of V12RAS larvae as measured by pigmented area, a combination of PD184352 or NVPBEZ235 with Rapamycin resulted in suppression of V12RAS-driven melanocyte hyperplasia to levels indistinguishable from wild-type larvae (Figure 5c and 5d). We next tested the ability of Rapamycin to co-operate with NVPBEZ235, in suppressing the growth of two established human melanoma cell lines in culture, WM1361 that like the zebrafish model harbour oncogenic RAS and A375 harbouring mutant BRAF. Using the Chou-Talalay method [13] and applying the CompuSyn software (ComboSyn, Inc.), combinatorial indices (CI) were calculated. It was shown by calculating the IC_50_ and the CI that there was a very strong synergy at very low (nanomolar) Rapamycin and NVPBEZ235 concentrations for these two cell lines as well as 501mel cells (Figure 5e-5g and Supplementary Figure S4).

### Further evaluation of the remaining hits displaying co-operation with MEKi or PI3K/mTORi

**Figure 6:**
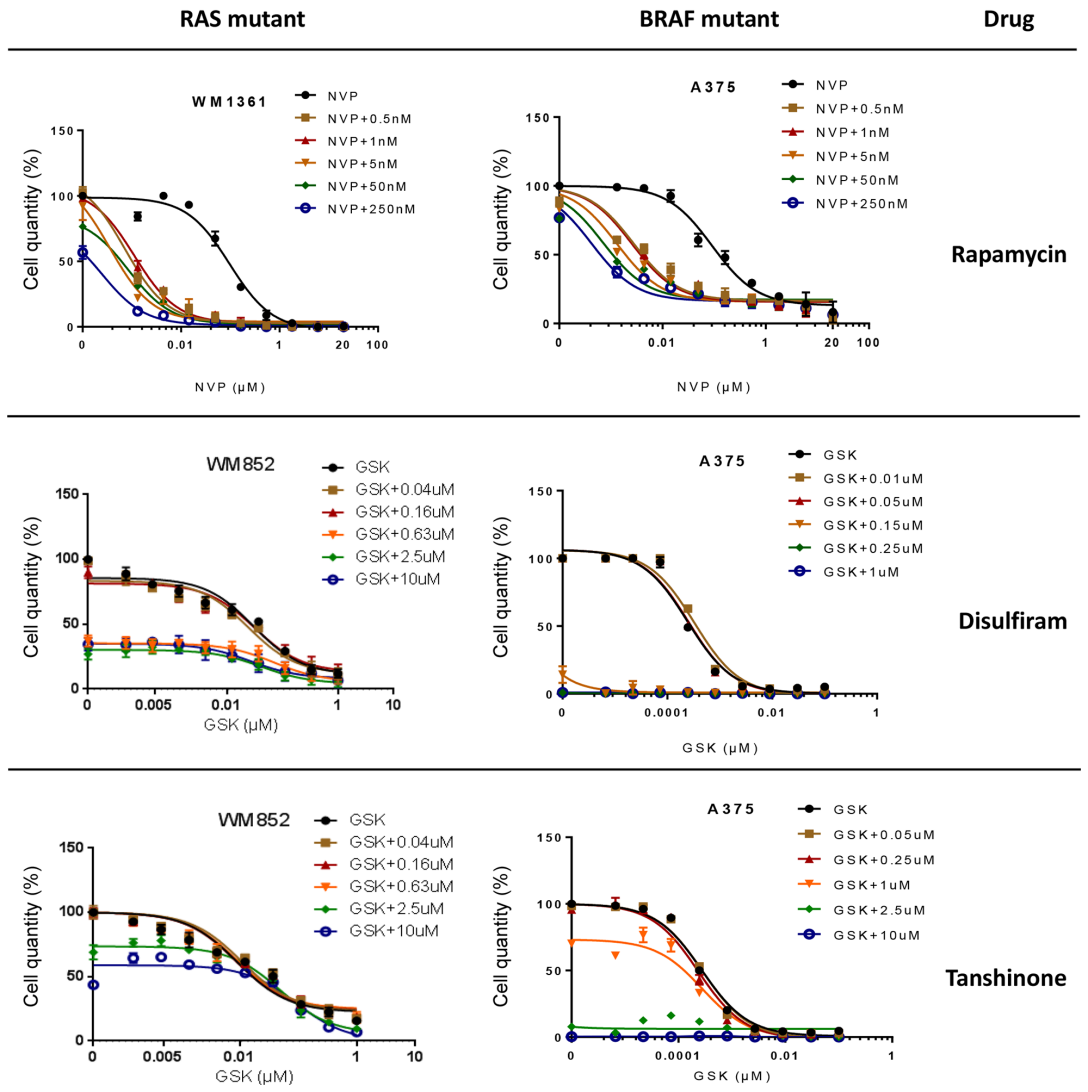
In-vitro effects of three selected hits Average cell number (%) is shown following 72h incubation with increasing concentrations of PD184352 (PD) or NVPBEZ235 (NVP) in the absence or presence of a fixed concentration of Rapamycin, Disulfiram or Tanshinone as indicated.

**Figure 7:**
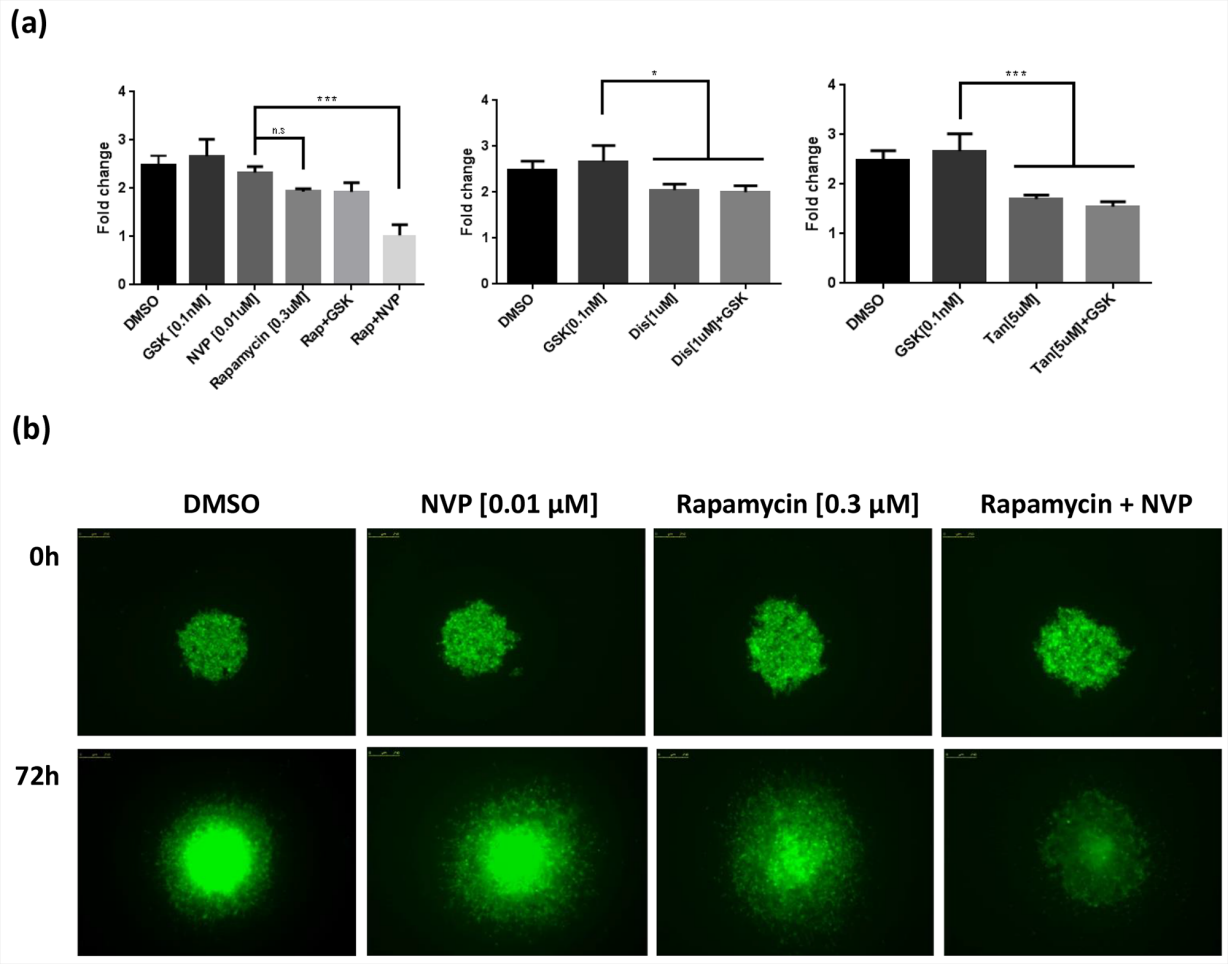
Hits effect on three-dimensional spheroid assay **a.** GFP-expressing A375 cells were allowed to form spheroids that were treated for 72h with Rapamycin (0.3 μM), Disulfiram (1 μM), or Tanshinone (5 μM). These drugs were also tested in combination with the inhibitors GSK1120212 (GSK) and NVPBEZ235 (NVP) at 0.1nM and 0.01 μM concentrations respectively. Quantification of the area of the spheroid core was used to establish a fold change ± SEM. *** p<0.001, ** p<0.01, * p<0.05, n.s. no significant Tukey's HSD test. **b.** Representative images of A375 spheroids treated with the synergistic combination of Rapamycin with NVP, and the appropriate controls, when the drugs were added (0 hours) and after 72 hours.

As for Rapamycin, the growth suppressive activities of the remaining 10 shortlisted hits were evaluated against established human melanoma cell lines that carry activating mutations in RAS (WM1361, MM485, WM852, and DO4) or BRAF (A375, 501Mel, and WM2664). GSK1120212, a more potent MEK1/2 inhibitor than PD184352 for human cells used in the clinic, was used as the representative MEKi. Of the remaining hits co-operating with PD184352 or NVPBEZ235 in our zebrafish screen, only Disulfiram and Tanshinone augmented the activity of GSK1120212 at concentrations <10μM (considered a selective concentration). However, in contrast to Rapamycin, only additive effects were observed (Figure 6 and Supplementary Figure S4). Potentially, drug responses of melanoma cells might be different when grown as 3D masses in extracellular matrix. To address this, we created spheroids of A375 cells and embedded them in a matrix of collagen I before exposing them to drug combinations. Again, we observed a super-additive antagonism of melanoma cell growth for the combination of Rapamycin and NVPBEZ235 but only an additive effect with combinations of GSK1120212 and Disulfiram or Tanshinone (Figure 7).

## DISCUSSION

The introduction of target-based drug discovery has coincided with a steady decline in R&D productivity in the pharmaceutical industry [14, 15]. 80% of candidate drugs fail by the end of Phase III, with clinical safety, lack of efficacy, poor bioavailability, or toxicity being the cause in approximately 70% of cases [16]. Problems are particularly acute in the field of cancer drug discovery, where the chance of a candidate drug obtaining regulatory approval following entry into Phase I studies is only 5% [16]. This implies that target identification and validation are currently extremely flawed exercises for many diseases, and that insufficient emphasis is given to optimizing drug pharmacokinetics and bioavailability during drug development. Target discovery for cancer is fraught with pitfalls. Due to the genomic instability acquired by cancer cells, a bewildering number of genes are mutated or differentially expressed in transformed cells compared to their normal counterparts. This makes distinguishing drivers that are integral to tumourigenesis from passengers very challenging. Consequently, drug companies pursue the same ‘low lying fruit’ (the handful of well-documented oncogenic drivers) resulting in a lack of diversity in the pharmaceutical portfolio. Innovative new strategies are required to complement existing techniques to increase success rates in cancer drug discovery.

An alternative to target-based drug discovery is the reintroduction of phenotype-guided discovery in the form of forward chemical genetics. This approach combines high-throughput screening with in vivo disease models. Large chemical libraries can be screened in whole organisms to identify entities that modify a disease phenotype, with the aim to produce efficacious, non-toxic hits early on in drug development. As this approach is not reliant on a previously defined target, it bypasses a major bottleneck in target-based discovery. This can also lead to the development of novel treatments and give insights into previously undefined targets and pathways contributing to disease. Further, compounds with poor pharmacology or toxicity will be excluded at the outset. Large-scale chemical screens have previously been restricted to invertebrate model organisms such as *Drosophila* and *C. Elegans* or to cell or tissue based assays. The logistics and expense involved in screening thousands of compounds in a vertebrate organism such as the mouse has limited its widespread adoption. Zebrafish are unique among vertebrate models in being amenable to high throughput chemical screens [7].

This work set out to develop and validate a cancer model in zebrafish that could feasibly be used in high throughput chemical screens to aid cancer drug and target discovery. A transgenic zebrafish melanoma model was developed by coupling the zebrafish *mitfa* promoter, which is highly selective for melanocytes, to human oncogenic HRAS^G12V^. V12RAS transgenic embryos developed melanocyte hyperplasia, which we could quantify using a simple measure of melanin content, accompanied by stimulation of RAS-RAF-MEK-ERK and RAS-PI3K-AKT-mTOR signalling pathways. The early onset and reproducibility of the V12RAS phenotype coupled to the sensitivity of the melanin content assay enabled the screening of small molecule inhibitors on V12RAS embryos to investigate their effect on neoplastic development. A number of selective MEKi and dual PI3K/mTORi inhibitors were shown to suppress V12RAS driven melanocyte hyperplasia, concomitant with suppression of ERK and AKT activation. Moreover, a combination of MEKi and PI3K/mTORi inhibitors was super-additive as previously observed for human melanoma cells [3–5]. This finding inspired a screen for compounds that might potentiate the activity of MEKi and PI3K/mTORi inhibitors, grounded in the premise that combination therapy is more effective and potentially less toxic than monotherapy. We sought these potentiators from among FDA approved compounds as existing drug libraries are enriched for biological activity and reprofiling/repurposing drugs is proving a remarkably efficient method of drug discovery [17], with notable examples in cancer drug discovery including Thalidomide, Metformin and Bosentan.

Screening a library of 640 compounds that are FDA approved, we identified 11 compounds (approximately 2%) which co-operated with either low dose PD184352 or NVPBEZ235 to suppress V12RAS-driven melanocyte hyperplasia without causing overt toxicity nor effecting pigmentation of wildtype animals. Among these hits, the mTOR inhibitor Rapamycin, originally developed for controlling transplant rejection, was the strongest potentiator, especially when combined with NVPBEZ235. Synergy of a Rapamycin analogue, or Rapalogue, Everolimus with NVPBEZ235 has previously been seen for hepatocellular carcinoma [18] and it was suggested that Rapalogues might improve access to the kinase active site of ATP competitive inhibitors. The ability of Rapalogues to co-operate with MEKi to suppress cancer cell growth and notably melanoma has also been previously observed [19]. Moreover, clinical trials of Rapalogues combined with MEKi or PIK3K/mTORi are currently underway.

While Rapamycin was the strongest potentiator identified in our modest screen, two other shortlisted compounds also showed activity both in fish and on cultured human cells: Disulfiram and Tanshinone. Antineoplastic effects of Disulfiram, an inhibitor of aldehyde dehydrogenases used to treat alcoholism, including toward melanoma have recently become known [20]. While a clinical trial of Disulfiram in melanoma as a monotherapy was unsuccessful, it might be worth re-evaluating its clinical potential in melanoma when combined with inhibitors of RAF-MEK-ERK signalling. Tanshinone (also called Danshinone) is a natural product from *Salvia miltiorrhiza* (red sage or Tanshen/Danshen) used widely in the Chinese medicine tradition for its anti-inflammatory and cardiovascular effects. Again antineoplastic activity has been reported including for melanoma [21] and from our findings Tanshinone might also be expected to co-operate with inhibitors of RAF-MEK-ERK signalling. Interestingly, Disulfiram and Tanshinone failed to show super-additive effects in cell culture but effects were super-additive in vivo (most notably for Disulfiram; Supplementary Figure S2b and Supplementary Figure S4). This observation could point to the ability of the molecules to not only target melanoma cell intrinsic mechanisms but also modify the pharmacology of either PD184352 or NVPBEZ235 in zebrafish (through for example modifying drug metabolism or excretion); or relate to aspects of disease mechanism, such as inflammation present in the larval disease model [22], which are absent in the more simplified human cell culture models. The remaining shortlisted hits did not appear to target melanoma cell intrinsic mechanisms, but by the above reasoning could influence drug pharmacology or extrinsic disease mechanisms. This reinforces the value of phenotype-guided drug discovery compared to target-based approaches. Further evaluation of the therapeutic potential of all 11 shortlisted hits would therefore be warranted in more relevant translational in vivo models, such as patient derived xenograft and mouse syngeneic allograft models.

## CONCLUSIONS

We provide proof-of-principle that V12RAS-expressing transgenic zebrafish embryos comprise an in vivo surrogate melanoma model that could feasibly be used to screen large chemical libraries for compounds that affect melanoma development, singly or in combination. The discovery of chemical modifiers of this phenotype could provide new insights into melanoma biology and new therapeutic drug leads.

## MATERIALS AND METHODS

### Animals

Zebrafish (*Danio rerio*) were raised and maintained at The University of Manchester Biological Services Unit under standard conditions as described elsewhere [23]. The wild type (WT) AB line was originally obtained from the Hubrecht Laboratory, and *Casper* strain (*roy*^−/−^, *nacre*^−/−^) zebrafish were a kind gift from Richard White (Harvard University). The transgenic line Tg(mitfa:V12RAS; mitfa:GFP) (herein named V12RAS) was previously described (Michailidou et al., 2009). All regulated procedures were approved by a local ethical review committee and performed under Home Office license.

### RT-PCR analysis

RNA was extracted from a pool of 30 wild-type and transgenic embryos at nine different time points ranging from 8hpf to 15dpf using TRIzol reagent (Thermo Fisher) according to the manufacturer's instructions. 1μg of each RNA sample was then used to synthesize cDNA using Omniscript RT kit (Qiagen). A minus RT enzyme reaction was also set up using a pool of either V12RAS or wild-type RNA to rule out sample contamination. The resulting cDNA samples were then used as template in PCR amplification reactions using the following specific primers:

RAS Fw 5′-GAACAACTGTGACCTGGCTG-3′

RAS Rev 5′-GAGGTGTGGGAGGTTTTTTAA-3′

BETA ACT Fw 5′-CGTGCTGTCTTCCCATCCA-3′

BETA ACT Rev 5′-GGTCTCGAACATGATCT GTG-3′

and standard cycling conditions for 28 cycles. A positive ‘housekeeper’ gene β-actin was also assayed for each sample in order to verify cDNA integrity and consistency between samples.

### Antisense oligonucleotide injections

Approximately 1 nl of 0.5mM morpholino-modified antisense oligonucleotides was injected into 1-cell stage larvae using a PI-100 pico-injector (Harvard Apparatus). V12RAS Oligonucleotide sequence was as follows:

5′- CTGGAACATCGTATGGGTAAGCCAT -3′;

V12RAS oligonucleotide was designed by and purchased from GeneTools. The standard control oligonucleotide from GeneTools was used as a control for injection artefacts.

### Histology and immunostaining of zebrafish larvae

Tissue sections (5 μm) from MS222 anaesthetized, PFA-fixed and paraffin-embedded larvae were used for Haematoxylin and Eosin (H&E) staining and Immunohistochemistry (IHC). H&E staining was automated (Thermo Shandon staining machine). Prior to IHC, tissue sections were bleached by incubating sections in 3% hydrogen peroxide diluted with 0.05M phosphate buffer at 55°C for 30 minutes. Sections were then washed and blocked with 5% normal goat serum/TBST for 1 hour at room temperature. Following the blocking step, sections were stained using rabbit anti-phospho-ERK (Cell Signaling Technology #4370S) and rabbit anti-phospho-AKT (Cell Signaling Technology #3787S) primary antibodies, as per manufacturer's instructions, followed by a biotinylated secondary horse anti-pan-immunoglobulin antibody (Vector Labs). Binding was revealed using an ABC kit with a DAB peroxidase substrate (Vector Labs). Pre-immunized rabbit serum was used in place of a specific primary antibody as a control for specific staining. Sections were scanned and images captured and processed using a Zeiss Mirax Scan system with a x20 objective lens f and a Marlin f14 6C camera.

### Larval drug treatments

Wild-type and V12RAS embryos were cultured in non-treated CytoOne 24-well plates (Starlab) from 2dpf until 5dpf with MEKi or PI3K/mTORi at the stated concentrations. A 0.1% DMSO treatment was used as a negative control. 10 embryos were placed per well in 2ml of buffered reconstituted de-ionized water (1 mM Tris pH 7.4, 6×10-3% w/v instant ocean (Tropic Marin), 5×10-5%w/v methylene blue hydrate (Sigma)) to which the appropriate molecules were added. These plates were incubated in a LMS incubator at 28°C and air atmosphere.

### Immunoblotting

Protein was extracted from MS222 anaesthetized 5-dpf larvae (n=30 for each sample) treated for 4h with DMSO alone or inhibitor and resolved in 4-12 % Bis-Tris Novex precast gels (Invitrogen) using XCell SureLock Mini-Cell tanks (Invitrogen). Proteins were transferred to Immobilon-P transfer membranes (Millipore), using a XCell II Blot Module (Invitrogen). Membranes were incubated with the following primary antibodies: anti-phospho-AKT (Cell Signaling Technology #2965), anti-total AKT (Cell Signaling Technology #9272), anti-phospho-ERK 1/2 (Cell Signaling #4370s), and anti-total ERK (Cell Signaling #9102). The secondary antibody was either anti-mouse immunoglobulin or anti-rabbit immunoglobulin conjugated to horseradish peroxidase (HRP) (Amersham Biosciences). Proteins were visualized with ECL plus (PERKin-Elmer) as per manufacturer's guidelines. Film was developed in a film processor (Optimax, IGP). Protein bands were quantified using ImageJ (public domain). All immunoblotting procedures were triplicated in entirety with equivalent results.

### Chemical library

A collection of 640 FDA approved small molecules were screened. An aliquot of the library (Enzo Life Sciences, Cat. No: BML-2841-0100) was supplied by Clemens Grabher (Karlsruhe Institute of Technology (KIT), Germany). All compounds in the library were dissolved in DMSO at an average 10 mM concentration. These drugs were tested initially at an average concentration of 1 μM in conjunction with either a MEK inhibitor (PD184352) or PI3K/MTOR inhibitor (NVPBEZ235), applied at a suboptimal concentration (0.1 μM and 0.3 μM respectively).

### Larval melanin content assay

After drug treatment, MS222 anaesthetized larvae were transferred to non-coated 96-well plate (eight larvae per well). The water was removed by careful aspiration and the wells were refilled with 200 μl of a fresh made solution of 1M NaOH. Plates were sealed and agitated at 37°C until animals were completely dissolved. Absorption was then measured at 340 nm in a 96-well plate reader (BIO-TEK Synergy HT). *Casper* values were subtracted to cancel out the signal from the eyes.

### Dorsal stripe melanocyte count and area determination

Larvae were treated with 5mg/ml epinephrine (Sigma) for five minutes to contract melanosomes, MS222 anaesthetized and then fixed in 4%w/v paraformaldehyde (PFA). One larva was then transferred to a watch glass with methylcellulose at room temperature. Images were acquired using a Zeiss Axiocam MRc camera mounted on a Zeiss Lumar V12 stereomicroscope. The number of melanocytes was established by manual counting and total area covered by melanocytes within the area of a standard 1000×500 square starting from the larval yolk sac extension was measured automatically using Image J software (http://imagej.nih.gov/ij/).

### Cell culture

501mel, A375 cells (purchased from ATCC) as well as WM852 cells (obtained from Wellcome Trust Functional Genomics Cell Bank, St George's Hospital Medical School), and WM1361, and MM485 cells (kind gifts of Claudia Wellbrock) were cultured in DMEM (Sigma) supplemented with 10% foetal calf serum (GIBCO) and 1% penstrep (Sigma). All cells were maintained at 37°C with 5% CO_2_.

### Cell quantity calculation

Cells suspended in 100 μl of culture medium were added at different concentrations, depending on the cell line, to the wells of a non-treated CytoOne 96-well flat-bottomed plate (Starlab). After 24 hours of incubation, 25 μl of culture medium with the combination of drugs mentioned above were transferred to wells and plates incubated for three days. Cells were then carefully washed with PBS and the supernatants discarded. They were fixed and stained with 100 μl/well of a 0.05%w/v crystal violet solution in 4% v/v formaldehyde for 15 minutes. Subsequently, plates were rinsed with water, and left for 20 minutes with 150 μl/well of 2% SDS solution and gentle shaking to solubilize the stain. The absorbance of each well was then measured at 595nm in a 96-well plate reader (BIO-TEK Synergy HT).

### Spheroid assay

4000 A375-GFP cells were suspended in DMEM containing 5% FBS and 1.5% methylcellulose (Sigma). 100 μl solution per well was transferred into a 96-well U-bottom plate. Plates were incubated at 37°C with 5% CO_2_ for 24 hours to allow spheroid formation. Spheres were then transferred into 0.8 ml bovine collagen (3.1 mg/ml stock, 1.7 mg/ml final concentration PureCol) solution per well in a 24-well plate. The vehicle control DMSO, drugs on their own and their respective combinations, were added for 72 hours in 0.4 ml/well of DMEM/10% FCS. Images were acquired once the collagen was set (considered as 0 hours) and after 72 hours of treatment using a Leica DM IL HC inverted microscope and FC340 Cooled Monocrome camera (Leica Microsystems).

### Statistical analysis

Throughout, graphed data is expressed as mean ± standard error of the mean (SEM). Each data set's descriptive statistics and distribution were analyzed using the explore function in SPSS software. Statistical differences between two groups were assessed using independent sample *t-test* (SPSS software, SPSS Inc.) and statistical significance was set to *P*<0.05. Differences between several groups were tested using a One-way ANOVA, followed by a post-hoc Dunnett test to compare different groups to a control (SPSS software) or a Tukey's HSD test (R software). Statistical significance was set to *P*<0.05.

## SUPPLEMENTARY FIGURES


