# Supplementary material for: Reprofiling using a zebrafish melanoma model reveals drugs cooperating with targeted therapeutics
Source: Oncotarget. 2016 May 26;7(26):40348–61. doi: 10.18632/oncotarget.9613 (PMC5130012; doi:10.18632/oncotarget.9613)
Supplement: Supplementary file 1 [file oncotarget-07-40348-s001.pdf]

Reprofiling using a zebrafish melanoma model reveals drugs cooperating with targeted therapeutics

SUPPLEMENTARY FIGURES

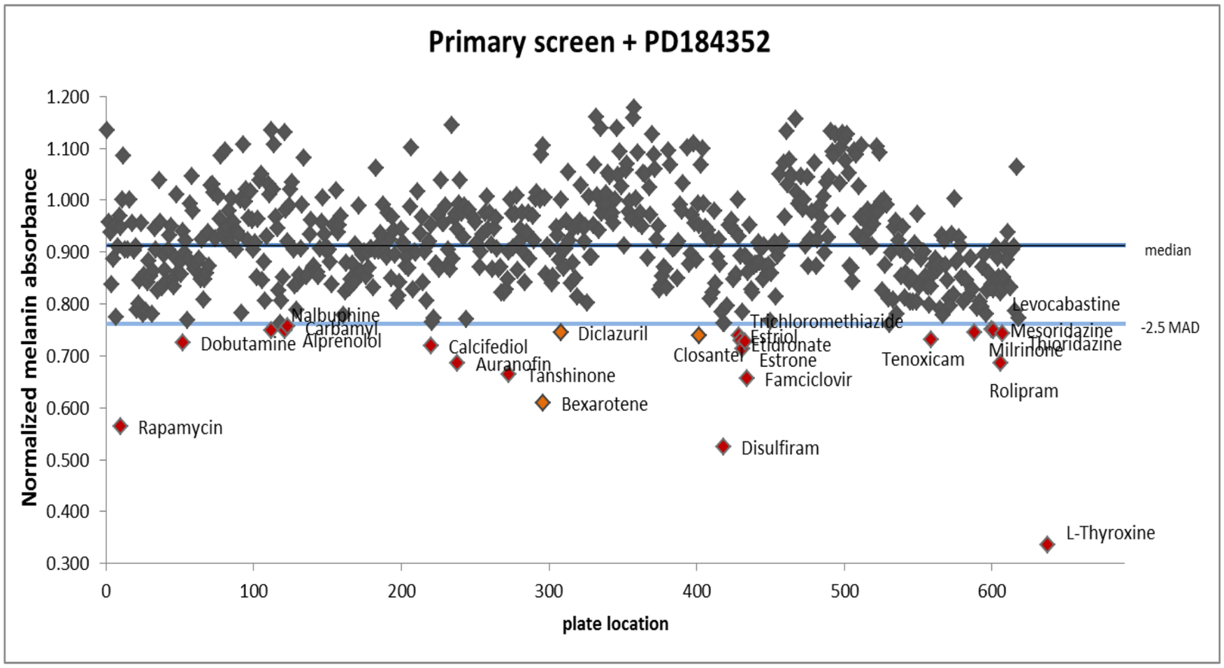

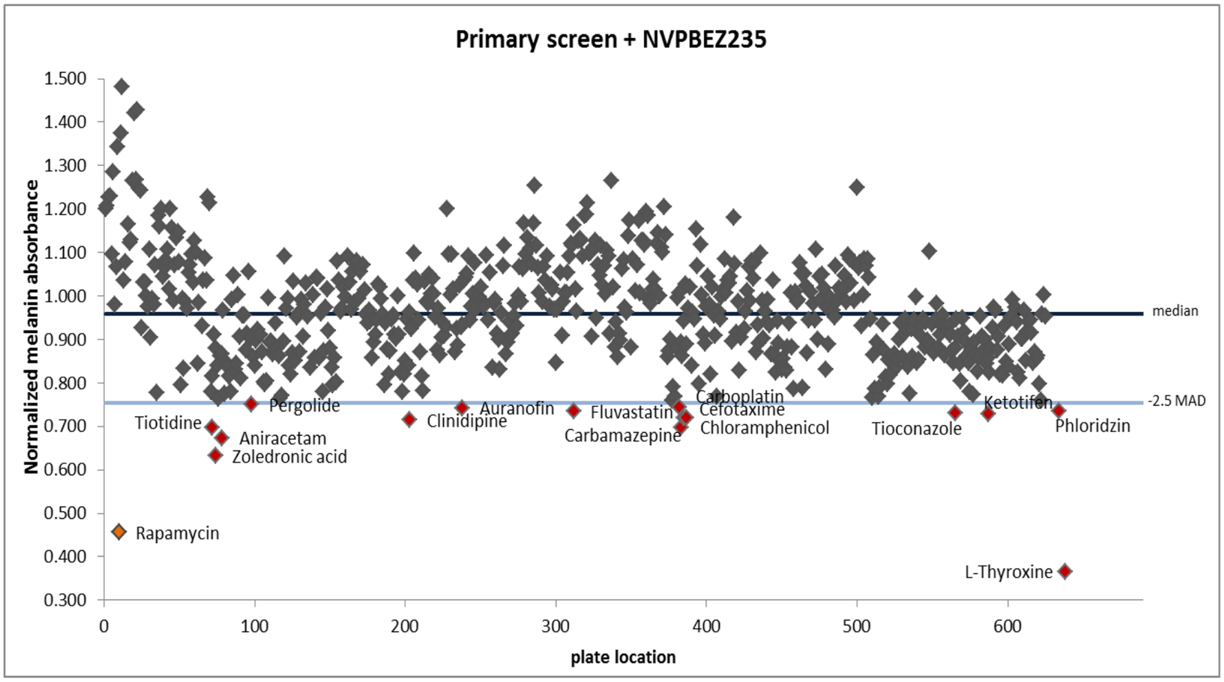

Supplementary Figure S1: Results and selection of hits from the FDA library screening. The 640 molecules from the FDA library were tested in combination with PD184352 (top) or NVPBEZ235 (bottom). The absorbance values from the melanin were normalized to DMSO-alone treated V12RAS transgenic embryos and plotted according to the plate location. The hits were selected considering the median and median absolute deviation (MAD) statistical method. The cut-off for hit identification was established at -2.5 MAD (blue line). Red dots represent hits from the primary screen and the orange dots are hits identified after halving the original drug concentration.

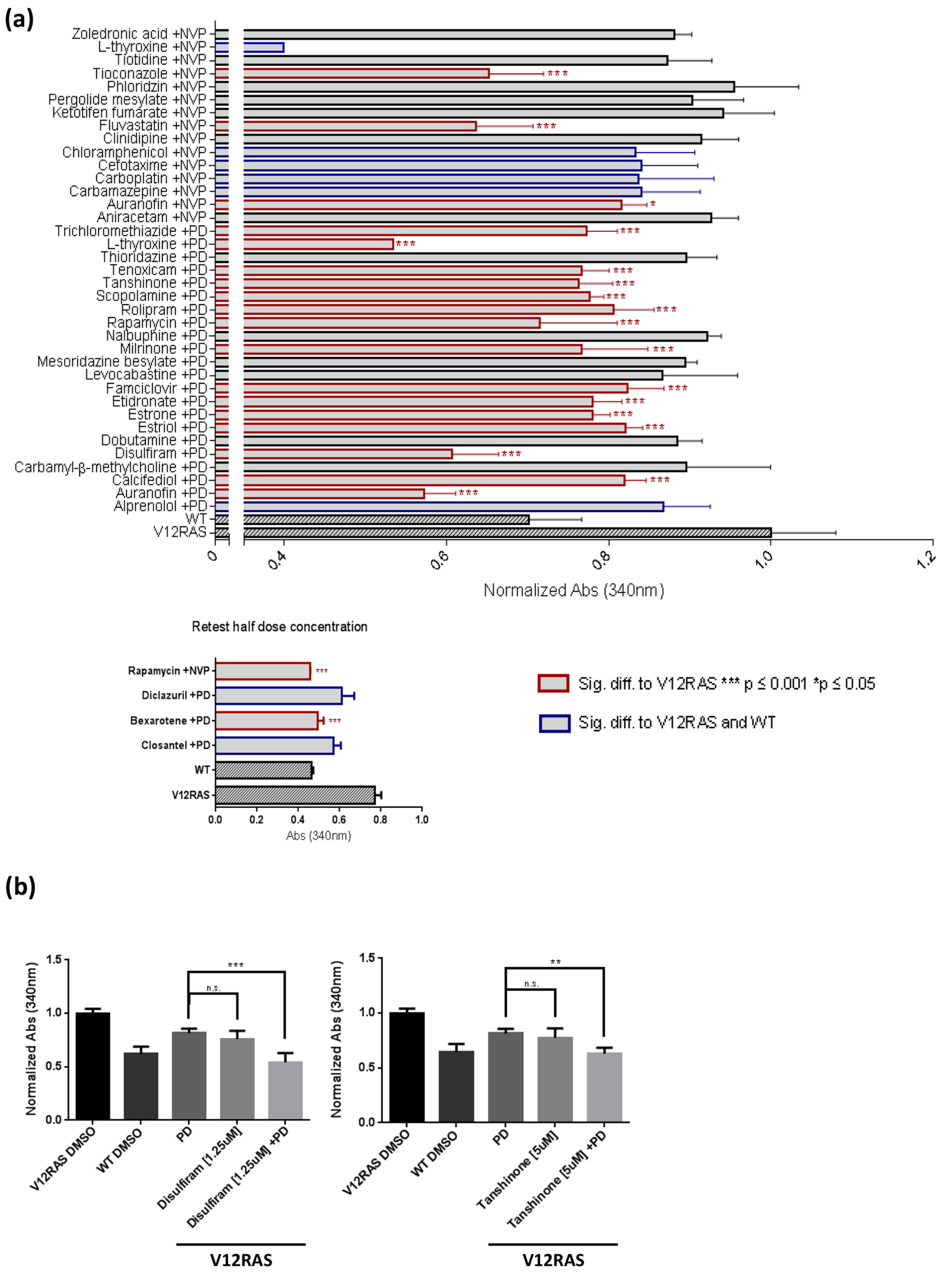

Supplementary Figure S2. Retest of hits from the primary screening. a. The 37 hits selected from the primary screen were retested to confirm their effect in combination with the appropriate inhibitor, compare to the values extracted from V12RAS transgenic embryos. Those hits that showed significant differences to V12RAS embryos (in red) or significant differences to both V12RAS and WT embryos (in blue) were considered for further testing (25 hits in total). Bars represent mean ± SEM. *** p<0.001, * p<0.05, Tukey's HSD test. **b.** Disulfiram and Tanshinone were tested using the larval melanin content assay at 1.25 µM and 5 µM respectively, as single agents and in combination with PD184352 (PD) (0.1 µM). WT and V12RAS embryos treated with vehicle control DMSO were used as reference values. The absorbance values from the melanin were normalized to DMSO-alone treated V12RAS transgenic embryos. Bars depicted mean ± SEM. *** p<0.001, ** p<0.01, Tukey's HSD test.

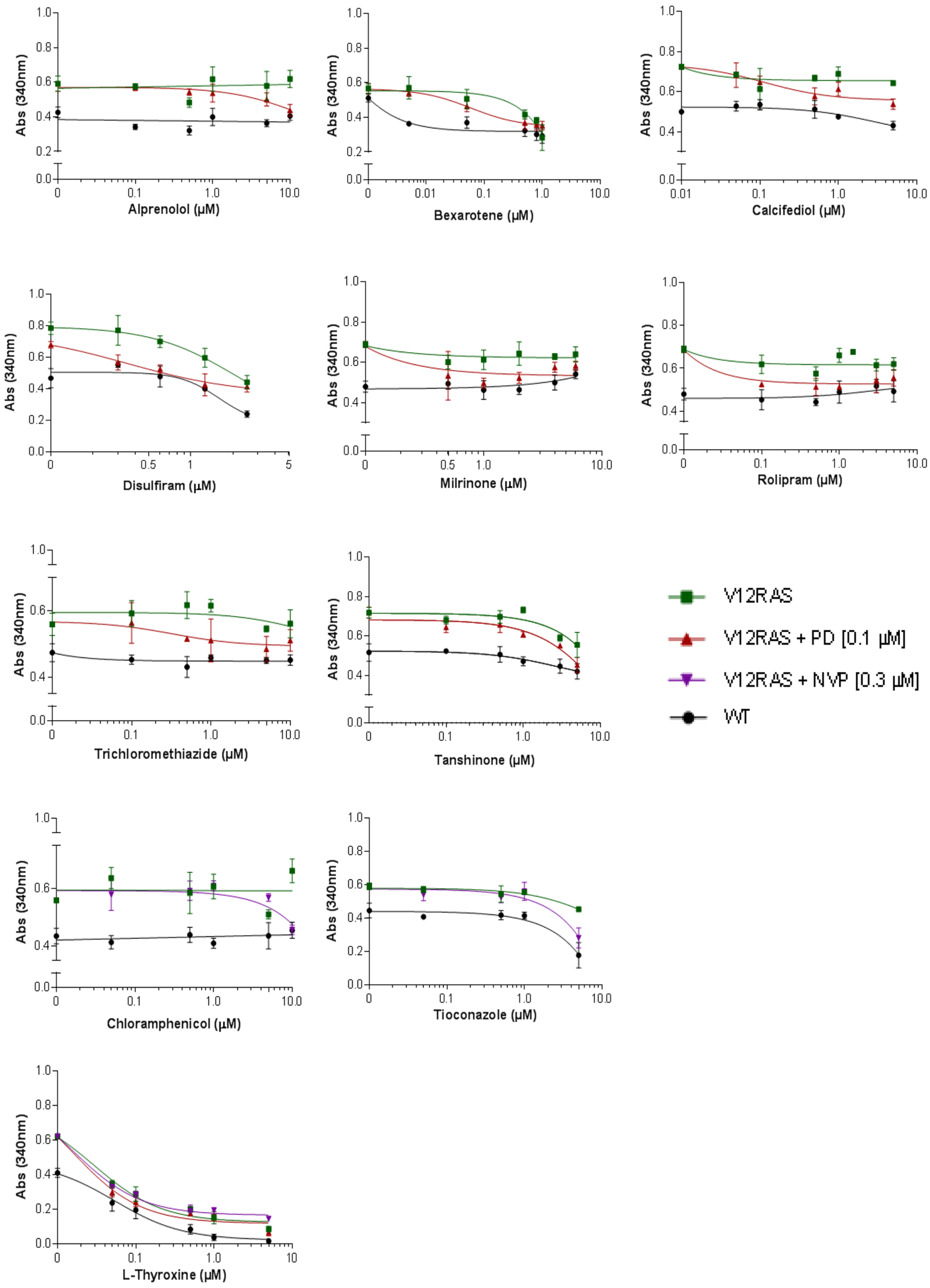

Supplementary Figure S3: Selected in-vivo drug dose response curves. Hit compounds were retested at different concentrations against V12RAS and WT larvae and for V12RAS transgenic emrbyos only in combination with either PD184352 (PD) or NVPBEZ235 (NVP) as appropriate. Points depict mean ± SEM for three independent experiments.

RAS mutant cells

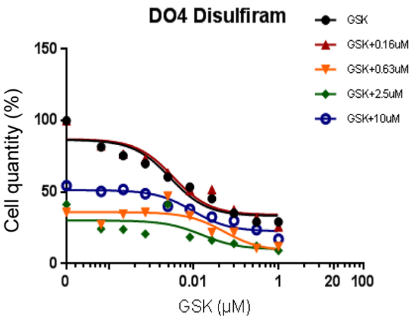

BRAF mutant cells

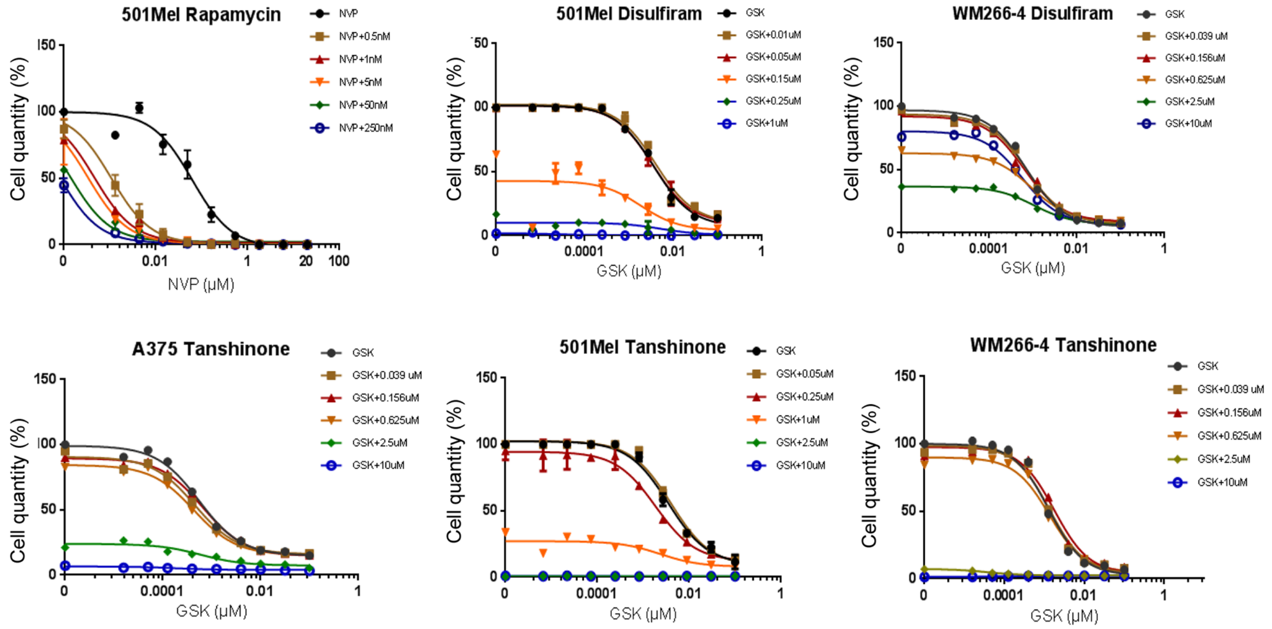

Supplementary Figure S4: Responses of other melanoma cells to selected hits. Average cell number (%) is shown following 72h incubation with increasing concentrations of PD184352 (PD) or NVPBEZ235 (NVP) in the absence or presence of a fixed concentration of Rapamycin, Disulfiram or Tanshinone as indicated.
